# Characterization of BPSS1521 (*bprD*), a Regulator of *Burkholderia pseudomallei* Virulence Gene Expression in the Mouse Model

**DOI:** 10.1371/journal.pone.0104313

**Published:** 2014-08-11

**Authors:** Sunisa Chirakul, Thanatchaporn Bartpho, Thidathip Wongsurawat, Suwimol Taweechaisupapong, Nitsara Karoonutaisiri, Adel M. Talaat, Surasakdi Wongratanacheewin, Robert K. Ernst, Rasana W. Sermswan

**Affiliations:** 1 Melioidosis Research Center, Khon Kaen University, Khon Kaen, Thailand; 2 Department of Biochemistry, Faculty of Medicine, Khon Kaen University, Khon Kaen, Thailand; 3 National Center for Genetic Engineering and Biotechnology (BIOTEC), Pathumthani, Thailand; 4 Biofilm Research Group, Faculty of Dentistry, Khon Kaen University, Khon Kaen, Thailand; 5 Department of Pathobiology, SVM, University of Wisconsin, Madison, Wisconsin, United States of America; 6 Department of Microbiology, Faculty of Medicine, Khon Kaen University, Khon Kaen, Thailand; 7 Department of Microbial Pathogenesis, School of Dentistry, University of Maryland-Baltimore, Baltimore, Maryland, United States of America; Tulane University School of Medicine, United States of America

## Abstract

The Gram-negative saprophytic bacterium *Burkholderia pseudomallei* is the causative agent of melioidosis, a severe infectious disease of both humans and animals. Severity of the disease is thought to be dependent on both the health status of the host, including diabetes mellitus and kidney disease, and bacterial-derived factors. To identify the bacterial factors important during an acute infection, gene expression profiles in the spleen, lung, and liver of BALB/c (Th2 prototype) and C57BL/6 mice (Th1 prototype) were determined using DNA microarrays. This analysis identified BPSS1521 (*bprD*), a predicted transcriptional regulator located in the type III secretion system (T3SS-3) operon, to be up regulated, specifically in C57BL/6 mice. BALB/c mice infected with a *bprD* mutant showed a shorter time to death and increased inflammation, as determined by histopathological analysis and enumeration of bacteria in the spleen. Elevated numbers of multinucleated giant cells (MNGCs), which is the hallmark of melioidosis, were detected in both the wild-type and the *bprD* mutants; a similar elevation occurs in melioidosis patients. One striking observation was the increased expression of BPSS1520 (*bprC*), located downstream of *bprD*, in the *bprD* mutant. BprC is a regulator of T6SS-1 that is required for the virulence of *B. pseudomallei* in murine infection models. Deletion of *bprD* led to the overexpression of *bprC* and a decreased time to death. *bprD* expression was elevated in C57BL/6 —as compared to BALB/c—mice, suggesting a role for BprD in the natural resistance of C57BL/6 mice to *B. pseudomallei*. Ultimately, this analysis using mice with different immune backgrounds may enhance our understanding of the outcomes of infection in a variety of models.

## Introduction

*Burkholderia pseudomallei* is a motile, aerobic, non-spore-forming, Gram-negative soil saprophytic bacterium [1]. It is the causative agent of melioidosis, an infectious disease endemic to Southeast Asia and northern Australia but present worldwide [2]. *B. pseudomallei* has been classified as a potential agent for bioterrorism (Tier 1 agent) by the U.S. Centers for Disease Control and Prevention, and has been suggested to be an emerging infectious disease [3]. In northeast Thailand, melioidosis accounts for 20% of community-acquired septicemias, with a 40% mortality rate even with appropriate treatment [4]. The clinical manifestations of melioidosis are diverse, and can include acute fulminant septicemia, localized lesions, and chronic disease. The disease is also associated with a high rate of relapse and affects all host organ systems [5]. The lungs are the most commonly affected organ, displaying primary lung abscesses or pneumonia followed by septicemia (blood-borne pneumonia) [1]. Although several virulence factors have been identified, the mechanism of pathogenesis is not fully understood. The ability of *B. pseudomallei* to invade, survive, and replicate intracellularly allows it to persist in the body during latent, chronic infection [6], [7], [8]. A number of virulence factors have been identified for *B. pseudomallei* infection of mammalian cells; these include type III (T3SSs) and type VI (T6SSs) secretion systems, quorum-sensing molecules, capsular polysaccharide, lipopolysaccharide, flagella, type IV pili, siderophores, and secreted proteins such as hemolysin, lipases and proteases [9], [10], [11].

As there is a broad spectrum of clinical outcomes and the severity of infection is associated with the diversity of the *B. pseudomallei* genome, the various virulence factors would expected to be differentially expressed under specific growth conditions. To identify and characterize bacterial genes whose products are involved in pathogenesis, two mouse strains with defined immunological backgrounds were used. The murine melioidosis models of acute (BALB/c: Th2 prototype) and chronic (C57BL/6: Th1 prototype) infections were used, as they have been shown previously to mimic the corresponding disease stages in humans [12]. BALB/c mice exhibit increased levels of proinflammatory cytokines, such as TNF-α, IL-1β, and IFN-γ, in addition to early infiltration of neutrophils, which contribute to the development of acute disease. Symptoms of acute disease include tissue destruction, multiple organ failure, and septic shock. In contrast, C57BL/6 mice, Th1 prototype, can effectively control *B*. *pseudomallei* infection, as demonstrated by moderate increases in cytokine levels and greater macrophage infiltration, allowing time for an adaptive immune response to occur [13], [14], [15], [16]. At present, the relative importance of the cell-mediated and humoral arms of the innate and adaptive immune responses is unclear [17], [18], [12].

In this study, *B. pseudomallei* 1909a, a highly virulent isolate from a severely septic patient, was used to establish an acute infection model in BALB/c and C57BL/6 mice. Gene expression profiles of bacteria replicating in the spleen, lung, and liver of mice at 4 days post-infection were compared with those of bacteria cultured *in vitro* using the whole genome *Burkholderia mallei/pseudomallei* DNA microarray from the Pathogen Functional Genomics Resource Center. Genes with unknown function and increased expression levels in at least two organs (lungs, spleen, or liver) of C57BL/6 mice were initially targeted for further analysis, as they may be associated with specific bacterial defense responses during acute infection.

## Results

### Expression of *bprD* is increased in the lungs and livers of C57BL/6 compared to BALB/c mice

To identify the bacterial factors important for acute *B. pseudomallei* infection, gene expression profiles in the lungs, livers, and spleens of BALB/c and C57BL/6 mice were determined. For this analysis, RNA was isolated at 4 days post-infection from the spleens, lungs, and livers of infected mice as well as from *B. pseudomallei*, 1909a cultured *in vitro* to the exponential phase.

Gene expression profiles determined using DNA microarrays obtained from the Pathogen Functional Genomics Resource Center (pfgrc.jcvi.org) showed that 5.0% of the *B. pseudomallei* genome (3.3% and 1.7% increased and decreased expression, respectively) in BALB/c mice, and 5.5% of the *B. pseudomallei* genome (3.3% and 2.3% increased and decreased expression, respectively) in C57BL/6 mice, were differentially expressed during acute infection (intraperitoneal injection), as compared to bacteria cultured *in vitro*. Genes with altered expression patterns were distributed equally between chromosome I—which encodes genes with core functions, such as metabolism and growth; and chromosome II—which encodes genes with accessory functions required for survival, and virulence factors [19].

**Figure 1:**
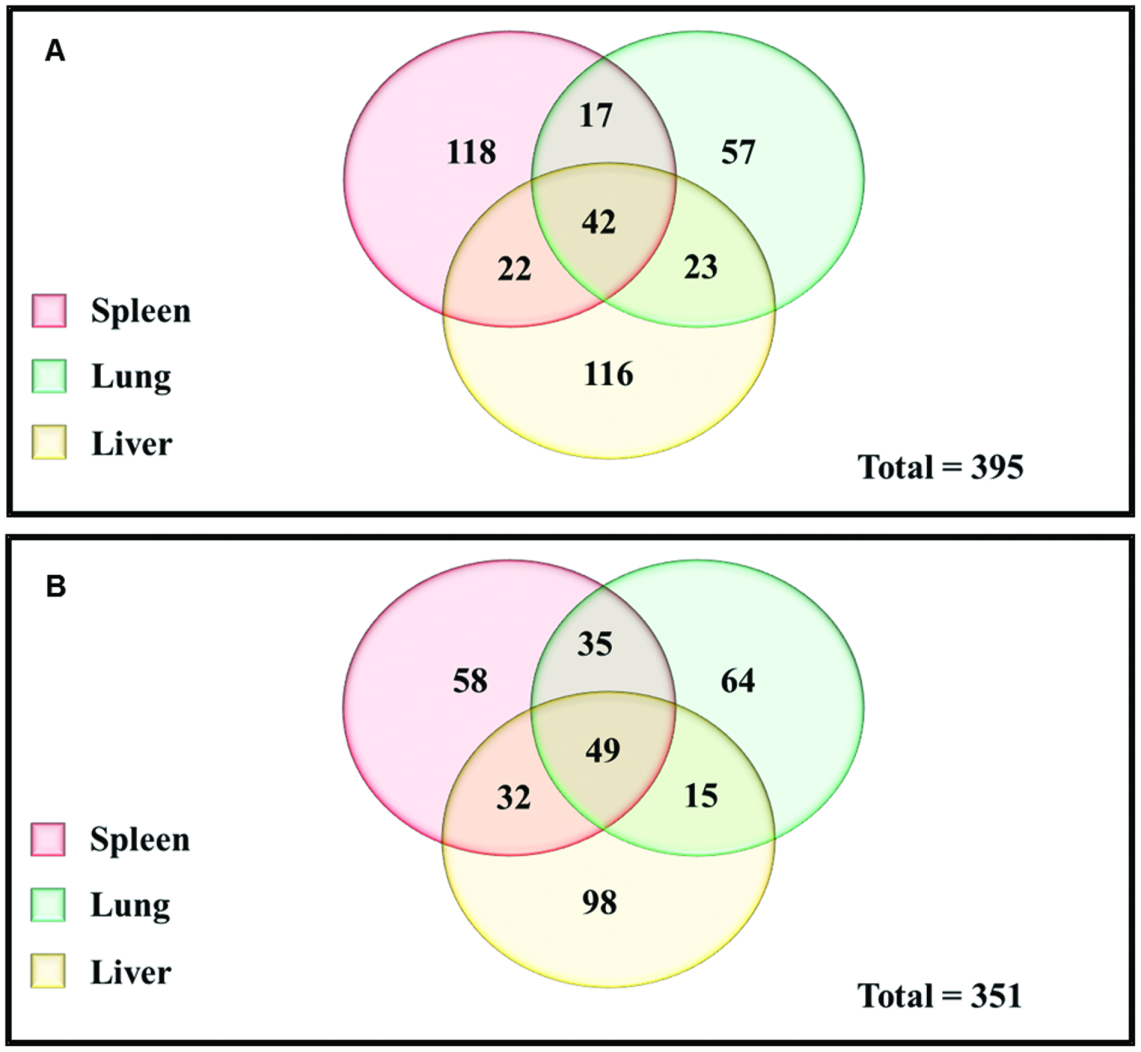
Venn diagrams showing the number of genes differentially expressed in each organ. Genes expressed at higher levels in the spleen (pink); lung (green); and liver (yellow) of BALB/C (A) and C57BL/6 (B) mice compared to *in vitro*.

**Figure 2:**
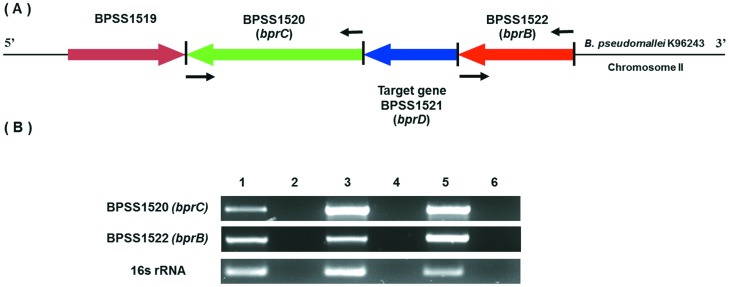
Schematic diagram of the *B. pseudomallei* K96243 *bprD* gene and agarose gel of RT-PCR products. Schematic diagram of the genomic organization of the *B. pseudomallei* K96243 region containing *bprD* (A), and RT-PCR analysis of expression of genes in this region (B). The arrows show the position and direction of genes. The positions of RT-PCR primers are indicated by black arrows. Lane 1, RT-PCR product from *B. pseudomallei* K96243 wild-type cDNA; lane 2, negative control for wild-type or DNase-treated wild-type RNA (to evaluate contamination of wild-type RNA with gDNA); lane 3, *B. pseudomallei* K96243 *bprD* mutant cDNA; lane 4, negative control for mutant or DNase-treated *bprD* mutant RNA (to evaluate contamination of mutant RNA with gDNA); lane 5, *B. pseudomallei* K96243 wild-type genomic DNA control; and lane 5, No DNA control.

To reduce the complexity of this dataset, genes whose expression was increased in all three of the target organs (lungs, liver, and spleen) were selected initially. This analysis resulted in: 1) 42 genes in BALB/c mice (Figure 1A); 2) 49 genes in C57BL/6 mice (Figure 1B); and 3) 34 genes whose expression was increased in both mouse strains. The majority of the genes in these three datasets were metabolic or housekeeping genes.

As this study aimed to identify novel *B. pseudomallei* genes important for virulence, the data analysis focused initially on genes of unknown function and those in known virulence operons, particularly the T3SSs and T6SSs operons [20]. This targeted analysis identified a total of 36 genes (Table 1), with 7 genes in T6SSs and 6 in T3SSs, the most interesting of which was BPSS1521 (*bprD*). *bprD* expression was 7.2-, 21.0-, and 18.6-fold, and 8.8-, 3.5-, and 3.5-fold, higher in the spleen, lung, and liver of infected C57BL/6 and BALB/c mice, respectively, as compared to bacteria cultured *in vitro*. Moreover, the fold changes in *bprD* expression in the lungs and livers of C57BL/6 mice were considerably higher than those in BALB/c mice (Table 1). Additional genes of interest were identified in the T6SS-1 operon, the most interesting of which was BPSS1512 (*tssM*). This gene showed higher expression levels in the lungs of infected C57BL/6 mice and the livers of infected BALB/c mice, as compared to bacteria cultured *in vitro*. *tssM* has been reported to suppress host immune responses during infection, thus increasing bacterial survival and decreasing the time to death of *tssM*-mutant-infected mice [21]. The expression patterns of both genes were validated by qRT-PCR. The *in vivo*/*in vitro* gene expression ratios of *B. pseudomallei* in the lungs of BALB/c and C57BL/6 mice were consistent with the microarray data (Figure S1).

**Table 1:** List of the *B. pseudomallei* genes that have >2SD fold change in expression (*in vivo/in vitro*).

Locus Tag	Gene Symbol (NCBI)	Gene description (NCBI)	Fold Change					
			Spleen		Lung		Liver	
			BALB/c	C57BL/6	BALB/c	C57BL/6	BALB/c	C57BL/6
BPSL0226	*fliJ*	Flagella fliJ protein	*6.33	*4.65	ND	ND	1.91	3.39
BPSL0274	*flgF*	Flagella basal body rod protein FlgF	*4.52	1.81	1.19	1.36	1.01	1.45
BPSL0029	*fliO*	Flagella protein	2.21	3.31	3.61	1.91	*13.55	2.7
BPSL0272	*flgD*	Flagella basal body rod modification protein	2.9	1.4	0.77	*9.58	0.79	1.81
BPSL0277	*flgI*	Flagella basal body P-ring protein	1.0	*8.26	2.23	*5.72	2.65	1.09
BPSL0808		Peptidase	1.2	1.82	1.5	*7.12	1.57	0.83
BPSL1505	*rpoS*	RNA polymerase sigma factor RpoS	1.42	1.56	*4.03	3.63	2.07	1.07
BPSL1819		Fimbriae-assembly-like protein	2.64	2.09	1.83	*4.43	2.55	2.6
BPSL1820		Fimbriae assembly-like protein	3.15	1.76	0.97	1.85	*4	3.27
BPSL1902		Hypothetical protein	2.52	1.42	*3.71	2.36	2.05	1.28
BPSL2403	*plcN*	Non-hemolytic phospholipase C	0.58	*7.9	0.9	2.21	3.4	0.43
BPSL2686	*rmlB*	dTDP-glucose 4,6-dehydratase	*3.9	1.04	2.28	2.54	1.57	1.18
BPSL3099		Outer membrane protein	0.84	0.88	0.98	0.98	*4.9	0.77
BPSL3172		Hypothetical protein	1.1	1.5	2.6	*4.3	0.78	2.74
BPSL3294	*flhA*	Flagella biosynthesis protein FlhA	0.68	0.66	0.36	1.28	*4.66	*3.89
BPSL3304	*tsr*	Methyl-accepting chemotaxis protein I	*3.91	2.49	0.78	*13.9	1.93	3.49
BPSS0029		Transport-related membrane protein	2.86	1.38	0.78	*6.12	0.67	*3.82
BPSS0417		Hypothetical protein	1.23	2.74	0.98	*6.15	1.24	*3.94
BPSS0520		Hypothetical protein	1.51	0.84	0.65	1.72	1.31	*6.03
BPSS0524		Hypothetical protein	1	*4.29	2.19	2.23	2.31	1.14
BPSS0860		Flagella hook-associated protein	1.41	2.14	1.29	*3.92	2.44	1.86
BPSS0989		Hypothetical protein	2.94	*3.75	1.71	2.46	3.09	1.65
BPSS1264		Hypothetical protein	2.62	2.35	*9.47	1.7	*94.6	6.24
BPSS1405	*sctS*	Type III secretion-associated protein	*3.92	2.16	1.47	2.08	1.93	3.49
BPSS1507	*ttssI*	Hypothetical protein	2.6	*3.79	0.75	2.24	1.13	*4.0
BPSS1512	*tssM*	Hypothetical protein	1.9	2.07	1.24	*7.36	*7.4	2.6
BPSS1521	*bprD*	Hypothetical protein	*8.8	*7.17	3.49	*21.0	3.46	*18.6
BPSS1524	*bopA*	Intercellular spread protein	0.45	0.52	*10.4	0.72	2.23	0.82
BPSS1531	*bipC*	Cell invasion protein	*3.89	3.35	*3.92	3.63	3.15	1.75
BPSS1533	*bicA*	Surface presentation of antigens protein	3.08	*4.6	2.51	2.66	2.47	2.527
BPSS1538	*bsaV*	Surface presentation of antigens protein	1.1	1.52	*3.82	1.05	*4.45	1.26
BPSS1543	*bsaQ*	Type III secretion system protein	1.25	*24.05	2.17	*8.19	2.63	0.83
BPSS1833	*udg2*	UDP-glucose 6-dehydrogenase 2	*4.17	1.15	1.84	1.9	1.44	1.07
BPSS1875		Chemotaxis-related protein	*3.84	*4.56	2.04	*7.54	1.4	3.49
BPSS2095		Hypothetical protein	2.45	1.59	0.42	*4.83	1.14	2.34
BPSS2103		Hypothetical protein	*6.74	1.65	3.5	*4.2	*4.0	2.28

* Expression level change >2SD in the organs of mice and ND  =  no data.

### BPSS1521 (*bprD*) mutation has no effect on growth rate *in vitro* but alters the expression of downstream genes

*bprD* (BPSS1521) is located in the T3SS-3 operon between BPSS1522 (*bprB*) and BPSS1520 (*bprC*). *bprB* and *bprC* are predicted to be response regulators; *bprC* has been reported to regulate T6SS-1 [9], [22]; however, the function of *bprD* is unknown [19], [23]. To investigate the function of *bprD*, a mutant strain was constructed using a double homologous recombination of a tetracycline cassette in *B. pseudomallei* strain K96243 [19], as the sequence of the 1909a strain is unknown. The K96243 strain is virulent in both BALB/c and C57BL/6 mice via the IP route of infection and is used commonly for cloning and investigation in numerous animal models [24], [25], [26], [27], [28]. The LD50 of the K96243 strain in BALB/c mice is 10^3^, compared to 20 for the 1909a strain. No significant differences in growth of the two *B*. *pseudomallei* strains were observed after *in vitro* culture in a rich medium (Luria–Bertani, LB) (Figure S2).

**Figure 3:**
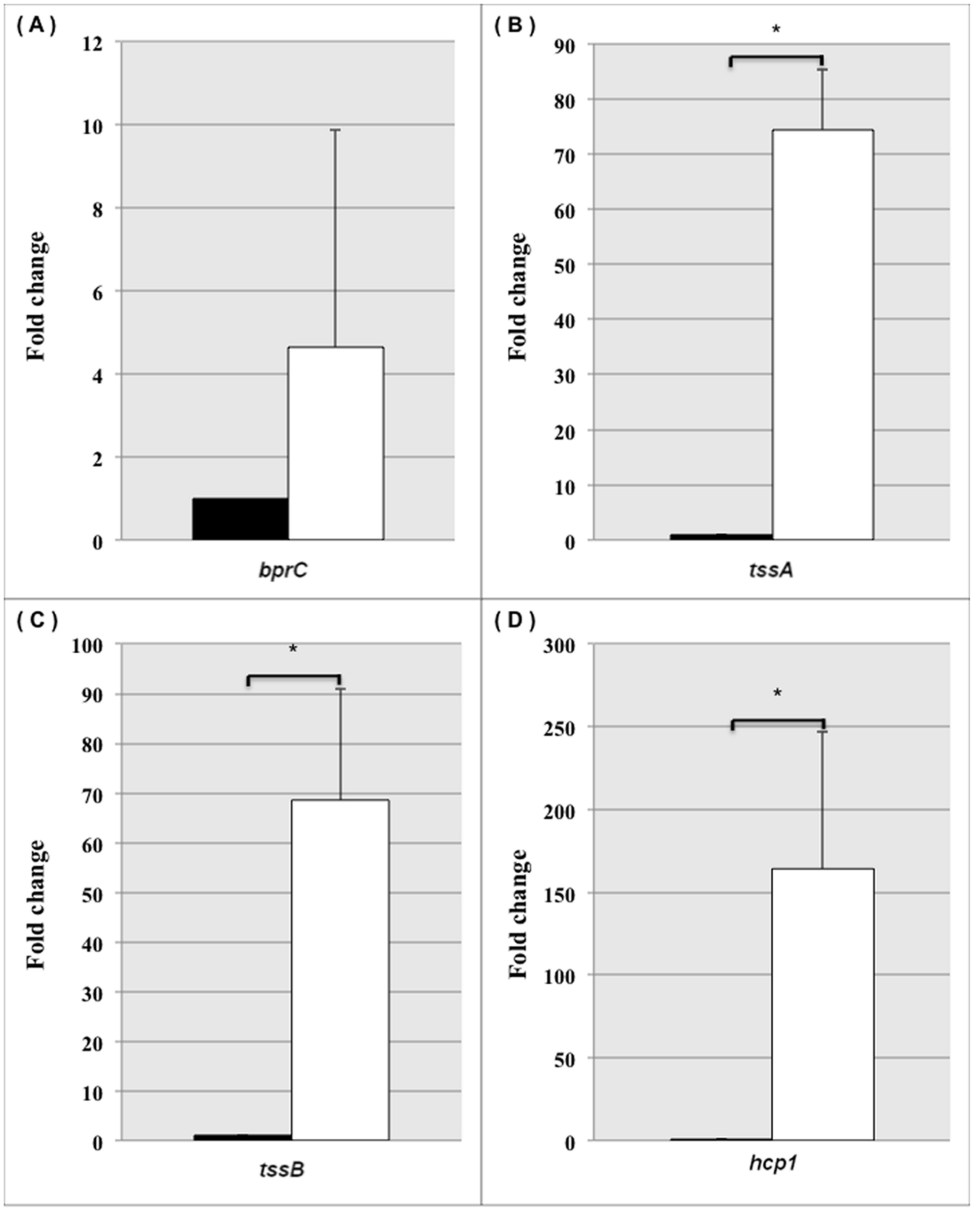
Fold changes in gene expression in the *B. pseudomallei* K96243 wild-type and *bprD*-mutant strains. The fold changes in expression in the *B. pseudomallei* K96243 wild-type (▪) and *bprD*-mutant (□) strains of the BPSS1520 (*bprC*) (A), BPSS1496 (*tssA*) (B), BPSS1497 (*tssB*) (C), and BPSS1498 (*hcp1*) (D) genes at mid-logarithmic phase in LB medium were measured by qRT-PCR. The bars indicate means ± standard error of two experiments; * significant difference.

As BPSS1521 is the second gene in the T3SS-3 operon, whether deletion of this gene altered the expression of the flanking genes, BPSS1520 and BPSS1522, was determined (Figure 2A). Expression levels of the individual genes were assessed using RT-PCR (Figure 2B) and qRT-PCR (data not shown). The results showed that *bprD* mutation did not affect the expression level of the upstream gene (BPSS1522 - *bprB*), but altered the expression of the downstream gene (BPSS1520 - *bprC*) (Figure 2B). BPSS1520 (*bprC*), an AraC regulator that is required for the expression of T6SS-1, was upregulated in the *bprD* mutant. To confirm upregulation of BPSS1520 in the *bprD* mutant strain, the T6SS genes, BPSS1496 (*tssA*), BPSS1497 (*tssB*), and BPSS1498 (*hcp1*), under the control of *bprC in vitro* [20] were analyzed using qRT-PCR. All T6SS genes were found to be expressed at higher levels in the *bprD* mutant (Figure 3).

Finally, to confirm that the insertion of the tetracycline marker in *bprD* did not result in a polar effect on *bprC*, a markerless allele replacement method was used to re-construct the *B. pseudomallei* K96243 *bprD* mutant [29]. Expression analysis in this mutant strain confirmed the increased expression of *bprC*, *tssA*, *tssB* and *hcp1*in the K96243 *bprD* mutant (data not shown).

### BPSS1521 (*bprD*) mutant results in a shorter time to death than the wild-type in BALB/c mice

**Figure 4:**
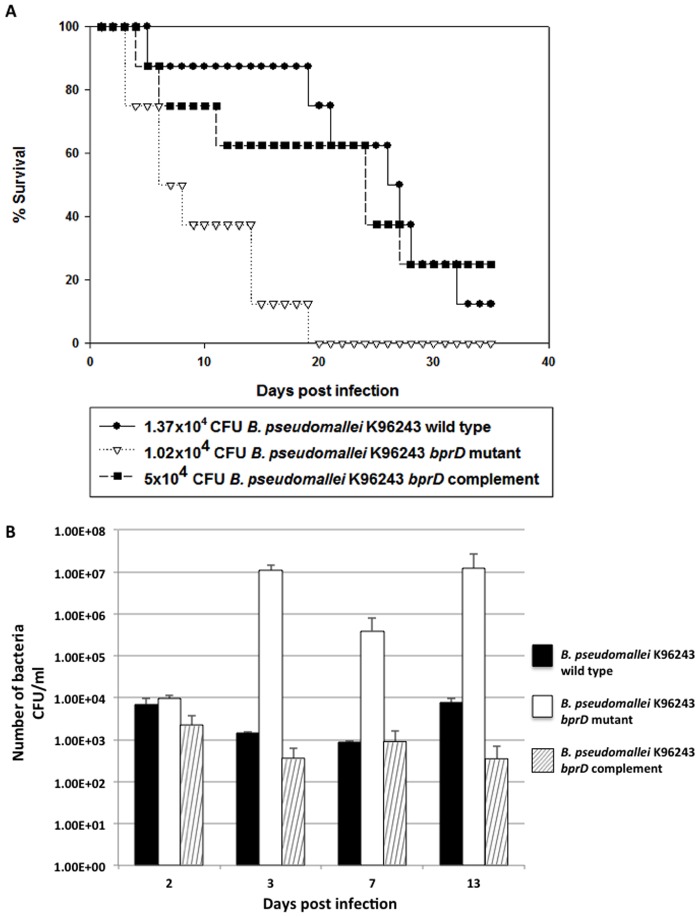
Survival curves and numbers of bacteria in the spleen of BALB/c mice after infection with *B. pseudomallei*. (A) The virulence of the *B. pseudomallei* K96243 wild-type and *bprD* mutant strains was compared in BALB/c mice, which are highly susceptible to infection. X-axis, days after infection; Y-axis, % survival. BALB/c mice (groups of eight) were intraperitoneally infected with ∼10^4^ CFU of the *B. pseudomallei* K96243 wild-type (•), *bprD* mutant (∇) or *bprD* complemented (▪) strain, and their survival was monitored daily. The survival of mice infected with the wild-type strain was significantly different from those infected with the *bprD* mutant (*P* = 0.0015). (B) The numbers of *B. pseudomallei* K96243 wild-type, *bprD*-mutant, and *bprD*-complemented strains in the spleen of BALB/c mice. X-axis, days after infection; Y-axis, number of bacteria (CFU). A total of 1×10^4^ CFU of the wild-type (▪) and *bprD*-mutant (□) strains, and 0.5×10^4^ CFU of the *bprD*-complemented ( 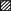) strain, were intraperitoneally injected into BALB/c mice. The number of bacteria in the spleen on days 2, 3, 7 and 13 was determined. The experiment was performed twice; average values of data are shown.

Both T3SS-3 and T6SS-1 are important for intracellular growth and survival. The T6SS-1 regulators (*bsaN*, *bprC*, and *virAG*) are essential for *B. pseudomallei* virulence in mice [20]. The virulence of the *bprD* mutant in mice was therefore evaluated. Because C57BL/6 mice are relatively resistant to *B. pseudomallei* infection and mimic chronic outcomes, while BALB/c mice are relatively sensitive and mimic acute infection, BALB/c mice were used for comparison of virulence. BALB/c mice, intraperitoneally infected with ∼10^4^ CFU of the *B. pseudomallei* K96243 *bprD* mutant showed a significantly shorter mean survival time (10 days), as compared to mice infected with wild-type K96243 (>25 days) (Figure 4A) (*p*<0.0015). Complementation with a functional copy of *bprD* (pBBR1MCS*bprD*) restored survival outcomes to wild-type levels (mean survival >21 days) (Figure 4A). The expression plasmid, pBBR1MCS*bprD*, in the *B. pseudomallei bprD* mutant was found to be stable throughout the course of the experiment (>21 days).

### BPSS1521 (*bprD*) mutant infection elicits an increased inflammatory response

As a splenic abscess is the most common complication of *B. pseudomallei* infection [1], bacterial growth and histological alterations resulting from *bprD* mutant replication in intraperitoneally infected BALB/c mice were evaluated. The bacterial burden in the spleen was similar at 2 days post infection with either the wild-type or the *bprD* mutant (6.9×10^3^ and 9.6×10^3^ CFU, respectively) (Figure 4B). Thereafter, the *bprD* mutant replicated rapidly, reaching 1.1×10^7^ CFU on day 3, and remained stable (∼1.2×10^7^ CFU) until day 13. In contrast, the burden of the wild-type strain decreased. Functional *bprD* complementation partially restored the behavior of the mutant strain (Figure 4B).

**Figure 5:**
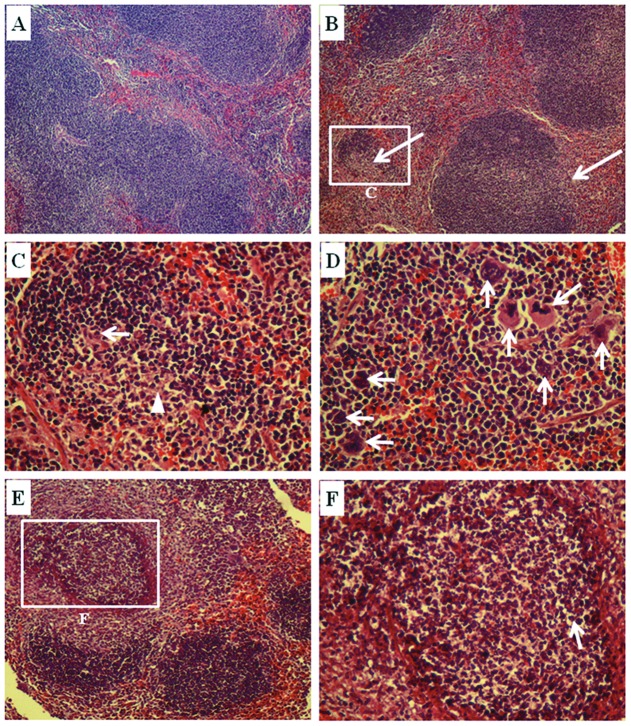
Photomicrographs of hematoxylin-and-eosin-stained spleens from BALB/c mice infected with *B. pseudomallei*. The spleens were collected on day 3 from non-infected BALB/c mice (A; ×100) and mice infected with *B. pseudomallei* (B–F). The spleen of mice infected with the *B. pseudomallei* K96243 wild-type strain showed multifocal areas of inflammatory cell infiltration and neutrophil abscess formation (arrows in B; ×100). The neutrophils (arrow) and necrotic cells (arrow head) are shown at high magnification (C; ×400). Numerous multinucleated giant cells are observed (arrows in D; ×400). Mice infected with the *B. pseudomallei bprD* mutant showed multifocal to coalescent pyogranulomatous splenitis (E; ×100). Necrotic areas with neutrophils (arrow) are shown at high magnification (F; ×400).

As the number of bacteria in the spleen at 3 days post infection differed significantly between the *bprD* mutant and the wild-type, a histological analysis of the spleens of infected mice was performed. The results indicated multifocal areas of inflammatory cell infiltration and neutrophil abscess formation (Figure 5B–C). Pathological scores were as follows: 1–4: 0  =  within normal limits; 1 = <25%; 2 = 25–50%; 3 = 50–75%; and 4 = >75% inflammatory cell infiltration and neutrophil abscess formation. The pathological score of spleens collected from mice infected with *B. pseudomallei* K96243 wild-type was 1.5, whereas that of mice infected with the *bprD* mutant was 3.0. Normal histology of spleens from non-infected BALB/c mice is shown in Figure 5A. Spleens from mice infected with either the wild-type (Figure 5D) or the *bprD* mutant displayed multinucleated giant cells. Spleens from mice infected with the *bprD* mutant showed multifocal areas of granulomatous reactions with necrotic centers (Figure 5E–F). These lesions contained a mixture of macrophages and neutrophils surrounding the central areas, which comprised necrotic cells and nuclear debris. Larger abscesses were found in mice infected with the *bprD* mutant (Figure 5E) as compared to the wild type (Figure 5B).

The liver pathology of mice infected with the *bprD* mutant differed only slightly from those infected with the wild type; however, micro-necrosis appeared to be more predominant in the livers of mice infected with the *bprD* mutant. No inflammation was observed in the alveolar spaces of lungs from both groups of mice at day 3 after infection (data not shown).

## Discussion

The pathogenesis of *B. pseudomallei* and how it interacts with and responds to the host immune system remain poorly understood. Both the host background and the diversity of bacteria contribute to the wide range of the disease outcomes. More than 50% of *B. pseudomallei*-infected patients are immunocompromised, with diabetes being the most prevalent underlying disease [30], suggesting that host immune status is important for the onset of disease. In this current study, *B. pseudomallei* gene expression profiles during acute infection in the spleen, lung, and liver of BALB/c (Th2-biased response, relatively susceptible to *B. pseudomallei* infection) and C57BL/6 (Th1-biased response, relatively resistant to *B. pseudomallei* infection) were assessed using whole-genome microarrays. The microarray data were in concordance with gene expression profiles from a hamster model of acute melioidosis [31] and a rat lung model of acute and chronic infection [32]. The majority of genes with altered expression levels (+/− 2SD compared with bacteria cultured *in vitro*) in both murine backgrounds in all three target organs (lungs, livers, and spleens) were related to metabolic function. The expression levels of a subset of T3SS-3 and T6SS-1 virulence factors were elevated in C57BL/6, as compared to BALB/c, mice. This suggests the influence of a strong Th1 response or a compensatory mechanism for bacterial survival.

T3SS-3 and T6SS-1 are essential for bacterial intracellular survival and contribute to the virulence of *B. pseudomallei* [20], [33]. Comparison of *B. pseudomallei* gene expression profiles in mice showed increased expression levels of BPSS1521 (*bprD*) in all target organs in C57BL/6 mice (7–21-fold changes), and in the lungs and livers of BALB/c mice (3–9-fold changes), as quantified by qRT-PCR.

*bprD* is located in the T3SS-3 operon between BPSS1522 (*bprB*) and BPSS1520 (*bprC*). BPSS1522 (*bprB*), BPSS1520 (*bprC*) and BPSS1521 (*bprD*) are located in the same operon, and BPSS1530 (*bprA*) is located downstream of these genes. Deletion of either *bprA* (Δ*bprA*::FRT) or all of *bprB*, *bprC* and *bprD* (Δ*bprBDC*::FRT) did not change T3SS-3 expression or secretion [22]. Chen and colleagues impressively uncovered the control cascade between T3SS-3 and T6SS-1 in murine RAW264.7 macrophages, and reported attenuated virulence of the *bprC*, *virAG* and *tssAB* mutants in BALB/c mice [20]. In this study, *bprC* expression was elevated in the *bprD* mutant, together with a shorter time to death, in BALB/c mice. Therefore, it was hypothesized that *bprD* acts as a negative regulator of *bprC*. Additionally, involvement of T6SS-1, which is required for intracellular growth, correlates with our findings of an increased bacterial burden in the spleens of mice infected with the *bprD* mutant at 4 days post-infection.

Pathological studies in BALB/c mice infected with wild-type *B. pseudomallei* showed numerous multinucleated giant cells (MNGCs), a hallmark of acute *B. pseudomallei* infection in cell culture [7] and in melioidosis patients [34]. It was shown previously that the T3/6SS components, *virAG*, *bprC* and *tssAB*, are required for MNGC formation. Our data indicate slightly increased numbers of MNGCs in the spleen of BALB/c mice infected with the *bprD* mutant, as compared to those infected with the wild-type. The *bprD* mutant showed increased areas of multifocal granulomatous reaction with necrotic centers, which contributed to a higher overall pathological score. The decreased expression of *bprC* due to suppression via BprD controls *tssAB* in T6SS-1, resulting in the establishment of a chronic infection in C57BL/6 mice. Further investigation is required to determine whether the Th1-biased immune response in C57BL/6 mice is involved in the altered expression levels of *bprD*. This phenomenon is similar to that reported for the *sseL* mutant in *Salmonella typhimurium* and *tssM* mutants in *B. pseudomallei* KHW [21], [35]. *tssM* (BPSS1512) is located in the T6SS-1 (BPSS1496-BPSS1512) operon of *B. pseudomallei* [36], and was reported by Tan and colleagues to play a role in suppressing the host innate immune response. Mice infected with the *B. pseudomallei* KHW *tssM* mutant showed a reduced overall time to death and an increased inflammatory response in the spleen [21].

In summary, the negative regulation of *bprC* by BprD sheds further light on the complexity of regulation between T3SS-3 and T6SS-1, and suggests that further investigation of the possibility of suppressing T6SS-1—which is the most important *B. pseudomallei* virulence factor —is warranted.

## Materials and Methods

### Ethics Statement

BALB/c and C57BL/6 mice were obtained from the National Laboratory Animal Center, Mahidol University, Bangkok, Thailand. The maintenance and care of the experimental animals complied with the National Animal Center guidelines (polycarbonate cages with stainless steel wire-bar lid feeders in which food is provided *ad libitum*, sterile wood shavings as bedding material, fed a commercial diet (No. CP 082; Perfect Companion Group Co. Ltd., Thailand). Reverse-osmosis (RO) drinking water was provided *ad libitum* and contained 10–12 ppm chlorine. The animals were maintained under a 12∶12-h light: dark cycle. The *B. pseudomallei* mouse infection protocol was approved by the Animal Ethics Committee, Khon Kaen University, Khon Kaen, Thailand (Reference No. AE06/53) based on the ethics of animal experimentation guidelines of the National Research Council of Thailand. All surgeries and terminations were performed under isoflurane anesthesia, and all efforts were made to minimize suffering. All clinical bacterial isolates were anonymized.

### Bacterial Strains, Plasmids, and Growth Conditions

Bacterial strains and plasmids used in this study are listed in Tables 2 and 3. The *B. pseudomallei* K96243 wild-type, *bprD* mutant, and *bprD* complemented strains, and the *Escherichia coli* strain used for construction of the *B. pseudomallei* mutant and expression strains were cultured in Luria–Bertani (LB) agar or broth with shaking at 200 rpm overnight. When necessary, antibiotics were supplemented as follows (in µg/ml): for *E. coli*, ampicillin (Ap), 100; chloramphenicol (Cm), 30; tetracycline (Tc), 25; and for *B. pseudomallei*, Cm, 30; Tc, 50. The requirement for approval of the use of all clinical isolates in a study of this nature was waived by the Human Ethics Committee of Khon Kaen University.

**Table 2:** Bacterial strains used in this study.

Bacterial strain			Description	Source or Reference
***B. pseudomallei***	1909a		Clinical isolate from sputum of 34-year-old male with diabetes mellitus who is a rice farmer in Ubonratchathani province, Thailand	Kindly provided by Dr. Narisara Chantratita, Mahidol Oxford Tropical Medicine Research Unit (MORU), Faculty of Tropical Medicine, Mahidol University.
***B. pseudomallei***	K96243		Clinical isolate from a patient admitted to Khon Kaen provincial hospital, Khon Kaen, Thailand	[21]
***B. pseudomallei***	K96243	*bprD* mutant	K96243 derivative; D*bprD*; Tc^r^	This study
***B. pseudomallei***	K96243	*bprD* complemented	*bprD* mutant carrying pBBR1MCS*bprD*; Tc^r^ Cm^r^	This study
***B. pseudomallei***	K96243	*bprD* clean mutant	K96243 derivative; D*bprD*	This study
***E. coli***	DH5α		General cloning	[46]
***E. coli***	SM10lpir		Mobilizing strain, SM10 with a l prophage carrying the gene encoding the π protein; Cm^s^ Tc^s^ Gm^s^ Km^r^ Tp^s^ Px^s^	[47]
***E. coli***	S17-llpir		Mobilizing strain, S17-1 with a l prophage carrying the gene encoding the π protein; Cm^s^ Tc^s^ Gm^s^ Km^s^ Tp^r^ Px^s^ Sm^r^	[49]

Cm, chloramphenicol; Gm, gentamicin; Ap, ampicillin; Tc, tetracycline; Km, kanamycin; Tp, Trimethoprim; Px, polymyxin; Sm, streptomycin; r  =  resistance; s  =  sensitive.

**Table 3:** Plasmids used in this study.

Plasmids	Description	Source or Reference
**pDM4**	Suicide vector; *sacBR* oriT oriR6K Cm^r^	[48]
**pDM4Δ** ***bprD***	pDM4::Δ*bprD B. pseudomallei* K96243; Cm^r^	This study
**pDM4Δ** ***bprD *** **::** ***Tc^r^***	pDM4::Δ*bprD*::Tc^r^ *B. pseudomallei* K96243; Cm^r^ Tc^r^	This study
**pBBR1MCS**	A Cm^r^ bhr plasmid of 4.7 kb, and contains 16 unique cloning sites within the lacZ∼ gene, Km^r^, Ap^r^, Tc^r^, and Gm^r^	[38]
**pBBR1MCS** ***bprD***	pBBR1MCS harboring the *bprD* gene	This Study
**pUTminiTn5Tc**	R6K-based suicide delivery plasmid; Tc^r^	Kindly provided by Prof. Ben Adler

Cm, chloramphenicol; Gm, gentamicin; Ap, ampicillin; Tc, tetracycline; Km, kanamycin; Tp, Trimethoprim; Px, polymyxin; Sm, streptomycin; r  =  resistance; s  =  sensitive.

### Biosecurity aspects

Concerning biosecurity aspects, both animal and general bacterial laboratory facilities were operated following all the security and safety regulations of our university. Animal experiments were carried out in the Northeast Animal Laboratory Center located in Faculty of Medicine, Khon Kaen University under the national procedure for infectious agents. This is a BSL2 plus facility that is currently being upgraded to BSL3 practices. Microbiological experiments were carried out under supervision of the director of the Melioidosis Research Center who received certificate of completion for BSL3 Executive Train-the-Trainer Program (Emory University, NSITE Applied Biosafety Training Program).

### Construction of the BPSS1521 (*bprD*) mutant and complemented strains

The genome sequence of *B. pseudomallei* 1909a is not yet available; furthermore, its high virulence prohibited comparison of its virulence with that of the mutant strain. Both 1909a and K96243 strains can infect and cause disease in BALB/c and C57BL/6 mice via the IP route. Therefore, the BPSS1521 (*bprD*) mutant was constructed in *B. pseudomallei* K96243 by double-crossover allelic exchange using the *λpir*-dependent pDM4 vector [48], and the constructed plasmids were introduced into *B. pseudomallei* K96243 by conjugation using the *E. coli* SM10*λpir* (kindly provided by Prof. Ben Adler, Monash University, Australia) as described by Milton in 1996 [37] and Songsri in 2012 [38]. Procedures for clone construction are described in Figure S4. The plasmids and primer sequences used in this study are listed in Tables 3 and 4. The clean deletion construct was confirmed by DNA sequencing.

**Table 4:** Primers used in this study.

GenBank accession number	Gene symbol	Gene Description	Primer name	Primer sequence	Purpose	Amplicon size (bp)
YP_111519.1	BPSS1512	Hypothetical protein	BPSS1512-F	GGACAAACGCTGGAAGTGAT	Internal region	129
YP_111519.1	BPSS1512	Hypothetical protein	BPSS1512-R	GTTCAGAAAGAACGCCTTGG	Internal region	129
YP_111526.1	BPSS1520	AraC family transcriptional regulator	BPSS1520-F	GGACGAGCTCGATTACATGC	Internal region	153
YP_111526.1	BPSS1520	AraC family transcriptional regulator	BPSS1520-R	GGCAGATGAAGATGCTGCTC	Internal region	153
YP_111527.1	BPSS1521	Hypothetical protein	BPSS1521-F	TCGATCTTCTCGCTGACCTC	Internal region	100
YP_111527.1	BPSS1521	Hypothetical protein	BPSS1521-R	TCAAGGAGATCCGCTTCAAC	Internal region	100
YP_111527.1	BPSS1521	Hypothetical protein	*bprD*-PF_Up_	TTAATTTCTAGAGCATCGGAGCAACAAGAATC	Upstream BPSS1521	927
YP_111527.1	BPSS1521	Hypothetical protein	*bprD*-PR_Up_	TTATGAAGATCTTTTCCTGTCGTGAACATTGG	Upstream BPSS1521	927
YP_111527.1	BPSS1521	Hypothetical protein	*bprD*-PF_Down_	TTGTCAAGATCTGCGTAACGTGTGACGTGTTG	Downstream BPSS1521	929
YP_111527.1	BPSS1521	Hypothetical protein	*bprD*-PR_Down_	ATAATACCCGGGGATGAAGATGCTGCTCGATG	Downstream BPSS1521	929
YP_111527.1	BPSS1521	Hypothetical protein	*bprD*-PF_Comp_	ATACTTGGTACCAGGAAAGACATCATGAAGCTC	Full-length BPSS1521	456
YP_111527.1	BPSS1521	Hypothetical protein	*bprD*-PR_Comp_	TTAACATCTAGATCACGGCGCCGGGCGCTG	Full-length BPSS1521	456
AY_305818.1		16S ribosomal RNA	16s rRNA-F	GGCTAATACCCGGAGTGGA	Internal region	194
AY_305818.1		16S ribosomal RNA	16s rRNA-R	CTAGCCTGCCAGTCACCAA	Internal region	194
YP_111503.1	BPSS1496	Hypothetical protein	BPSS1496-F	AGCGGGTCAACATCGTCTAT	Internal region	163
YP_111503.1	BPSS1496	Hypothetical protein	BPSS1496-R	ACGTCGTTGAAGTCGTCCTT	Internal region	163
YP_111504.1	BPSS1497	Hypothetical protein	BPSS1497-F	GCTCGCTGAAGTTTCTCGTC	Internal region	151
YP_111504.1	BPSS1497	Hypothetical protein	BPSS1497-R	TCGGCCGTATAGACCTTCTG	Internal region	151
YP_111505.1	BPSS1498	Hypothetical protein	BPSS1497-F	GTCATGACGGGAAAATCCAC	Internal region	134
YP_111505.1	BPSS1498	Hypothetical protein	BPSS1497-R	CGACGATCTGTCCATTTCCT	Internal region	134
pDM4			BAP3771	TAACGGCAAAAGCACCGCCGGACATCA	pDM4 MCS flanking region	177
pDM4			BAP3772	ACATGTGGAATTGTGAGCGGATAACAA	pDM4 MCS flanking region	177
		Tc^r^ cassette	BAP5118	GCGTAGTCGATAGTGGCTCC	Internal region	
		Tc^r^ cassette	BAP5116	ATCAGGGACAGCTTCAAGGA	Internal region	

MCS, multiple cloning site.

For complementation, the full length of *bprD* together with the ribosomal binding site (RBS) was amplified using *bprD*-PF_Comp_ and *bprD*-PR_Comp_ primers (Table 4), and then cloned into pBBR1MCS [39] at *Kpn*I and *Xba*I restriction sites to generate pBBR1MCS*bprD*. The constructed plasmids were then transformed into *E. coli* S17-1*λpir* and conjugated with the *B. pseudomallei* K96243 *bprD* mutant to generate the *B. pseudomallei* K96243 *bprD*-complemented strain.

The stability of pBBR1MCS*bprD* in the *B. pseudomallei bprD* mutant was investigated by culturing the *B. pseudomallei* K96243 *bprD* complemented with pBBR1MCS*bprD* in LB broth without antibiotics at 37°C overnight and sampling to LB agar plates daily for up to 21 days to obtain colonies. Ten colonies per day were PCR amplified using BPSS1521-F/BPSS1521-R primers (Table 4) to detect *bprD*.

The *B. pseudomallei* K96243 *bprD* clean deletion mutant was constructed by the allelic replacement system based on a non-replicative plasmid, pEXKm5 (kindly provided by Prof. Herbert P Schweizer, Colorado State University, USA), as described by Lopez in 2009 [29]. This mutant was used in the RNA-sequencing experiment to confirm the gene expression profiles of the *B. pseudomallei* K96243 wild-type and *bprD* mutant strains; this mutant will also be used for further study.

The *bprD* mutant and complement strain were originally constructed and examined for virulence. Subsequently, we constructed a clean deletion mutant confirming the role of bprD in virulence. Our initial animal studies clearly showed a role for this gene in virulence, therefore retesting the clean deletion mutant for altered virulence was not carried as it would replicate existing data and inappropriately increase the number of animals used.

### Animal infection and organ harvest

For the DNA microarray assay, BALB/c and C57BL/6 mice (males, 4–5 weeks old, 15–16 g, n = 15 mice each to be adequate for total RNA extraction) were intraperitoneally injected with 100-µl 1× PBS containing 10^3^ CFU of *B. pseudomallei* 1909a. Animals were then humanely euthanized with isoflurane, and the spleen, lung, and liver were harvested at day 4 post-infection (when bacterial numbers in the lung and liver are similar (Figure S3), and subjected to bacterial RNA isolation.

For virulence assessment, BALB/c mice, eight per group, were intraperitoneally inoculated with 100 µl of 10^4^ CFU/ml logarithmic-phase cultures of the *B. pseudomallei* K96243 wild-type, *bprD* mutant, or *bprD*-complemented strain. The experiment was performed independently in duplicate and viable bacteria were enumerated by the plate count technique. After injection, survival was monitored daily during 8:30 am–6 pm for 35 days, and mice showing severe signs of disease were euthanized for humane reasons when possible. After 35 days, the remaining animals were humanely euthanized with isoflurane.

### Histopathology and bacterial counts in spleen

Three groups of BALB/c mice, eight mice per group, were intraperitoneally injected with 10^3^ CFU of the *B. pseudomallei* K96243 wild-type, *bprD*-mutant or *bprD*-complemented strains. Two mice on each of days 2, 3, 7 and 13 were humanely euthanized with isoflurane, and bacteria in the spleens were enumerated. The average numbers of bacteria in the spleens from two independent experiments were reported.

For histopathology, two groups of BALB/c mice, three mice per group, were intraperitoneally injected with 10^3^ CFU of the *B. pseudomallei* K96243 wild-type or *bprD* mutant strain; 3 days later (when bacteria were present in the organs), they were humanely euthanized with isoflurane and a portion of the spleen, lung, and liver was fixed in 10% buffered formalin and embedded in paraffin. Serial 4-µm-thick sections from each organ were cut and placed on microscope slides. Dried slides were deparaffinized and stained with hematoxylin and eosin (H&E). The sections were visualized using a Nikon ECLIPSE 80i microscope and photographed with a Nikon DS-Fi1 digital camera (Nikon, Japan). Lesions were scored on the basis of inflammatory cell infiltration and neutrophil abscess formation using the scoring system described in the results section.

### RNA Manipulation

#### (a) Total RNA extraction from bacterial cultures

Total RNA from mid-log phase (0.4–0.5 OD_600 nm_) *B. pseudomallei* 1909a cultured in LB broth, at 37°C with shaking at 200 rpm was extracted using TRIzol Reagent (Invitrogen, USA), as recommended by the manufacturer. The RNA in DEPC-treated water was treated with RQ1 RNase-Free DNase (Promega, USA) to remove contaminating genomic DNA.

#### (b) Extraction of bacterial RNA from the organs of infected mice

The organs of infected BALB/c and C57BL/6 mice (spleen, lungs, and liver) (n = 15 from each mouse) were harvested, five mice at a time, under sterile conditions, transferred immediately to RNA*Later* (Ambion, USA), and chilled on ice. Each organ was rapidly homogenized separately in TRIzol reagent by grinding and sieving through a sterile stainless-steel sieve device, and then RNA was extracted as recommended by the manufacturer. The total RNA used for DNA microarrays was extracted from two independent groups of animals.

### cDNA synthesis

Total RNA extracted from each mouse organ during infection with *B. pseudomallei (in vivo)* or during the exponential phase of bacterial culture (*in vitro*) was converted to cDNA. In brief, 15 µg of RNA were mixed with 10 µg of Genome Direct Primers (bpGDPs) (1 µg/µl) to prime the transcription reactions, rather than using random primers to preferentially transcribe bacterial mRNA in the mixed RNA sample [40]. The mixture was heated at 70°C for 5 min, immediately chilled on ice and spun briefly to bottom down the solution. Then, 5 µl of 10 mM dNTPs, 5 µl of 5× first-strand buffer, 25 units of Recombinant RNasin Ribonuclease inhibitor, 1 µl of M-MLV RT (200 units/µl), and nuclease-free water were added to a 20-µl final volume. The mixture was mixed gently and incubated at 37°C for 60 min before being heat-inactivated at 70°C for 15 min. Unincorporated nucleotides were removed by gel filtration (Illustra MicroSpin G-50 Columns, GE Healthcare Bio-Sciences, NJ, USA).

### DNA labeling and microarray slide hybridizations

The whole-genome DNA microarray slides of *B. mallei* and *B. pseudomallei* version 2.0, received from the Pathogen Functional Genomics Research Center, MD, USA, were used in this study [41]. The gDNA of *B. pseudomallei* K96243 (10–15 µg) was labeled with Cy3-dCTP fluorescent dye by the standard nick translation reaction according to the manufacturer's protocol (Promega, WI, USA) [42]. For cDNA labeling, Cy5-labeled was incorporated using the Pronto Universal Hybridization kit (Corning, NY, USA), as described in the manufacturer's manual.

For hybridization, the genomic DNA was used to normalize each transcript signal to improve the signal-to-noise ratio [43]. Four micrograms of Cy5-labeled cDNA from each sample were mixed with 2 µg of Cy3-labeled gDNA of the K96243, dried in a SpeedVac concentrator for 30 min, and re-suspended in 50-µl Corning hybridization solution (Corning, NY, USA). Post-processed slides were hybridized while the LifterSlip Microarray Coverslips (Erie Scientific, USA) were placed over the array slides, incubated at 42°C in a water bath for 16–20 h, and post-hybridization washed according to the protocol recommended by Corning before scanning. DNA microarray slides, after hybridization and washing, were scanned by a GenePix 4000B laser scanner (Molecular Devices, Sunnyvale, CA, USA) and initially analyzed using the GenePix Pro 6.1 software to determine the fluorescence intensities of the two dyes for each spot.

### Microarray data analysis

The data analysis was performed as described by Bartpho et al., 2012 [42]. Hybridization signals from cDNA samples were compared to the gDNA hybridization signals to estimate the relative gene expression levels for normalization (genomic normalization) [43]. Data from replicate genes were averaged before gene identification. An average of two replicate hybridizations from two independent biological replicates was used to calculate gene expression levels. The log_2_ expression values *in vivo* and *in vitro* were used to identify those genes with mean log_2_ [*in vivo*]/[*in vitro*] ratios that deviated more than an overall mean of ±2 SD as being significantly differentially expressed *in vivo. B. pseudomallei* gene expression profiles in the lungs, spleens, and livers of BALB/c and C57BL/6 mice were performed as hierarchical clustering as groups of similarity in gene expression using a free software package [44], and mapped to COG functional categories [45].

The microarray data have been deposited in the NCBI Gene Expression Omnibus [46]. All data are MIAME compliant (Minimum Information About a Microarray Experiment) and gene expression profiles are accessible through GEO Series accession number GSE51369.

### Gene validation (qRT-PCR)

The selected genes were validated by quantitative real-time RT-PCR (qRT-PCR) using the LightCycler FastStart DNA Master^PLUS^ SYBR Green I with the LightCycler Carousel-Based System (Roche Diagnostics, USA). The specific primers for all selected genes, BPSS1520, BPSS1521, BPSS1522, BPSS1512, BPSS1496, BPSS1496 and 16s rRNA (an internal control) are listed in Table 4. All data were analyzed using the 2^−ΔΔCT^ method. The results were expressed as target/reference ratios of each sample divided by the target/reference ratio of the calibrator. When the target samples were the *B. pseudomallei* genes expressed *in vivo*, the target calibrators were the *B. pseudomallei* genes expressed *in vitro* and the reference was the 16s rRNA gene. Results were reported as means of triplicate samples with standard deviations.

## Supporting Information

Figure S1 **Fold changes in gene expression** ***in vivo*** **/** ***in vitro*** **.** Fold changes in the expression of the BPSS1521 (A) and BPSS1512 (B) genes as determined by DNA microarray (upper) and validated by qRT-PCR (lower) in the lungs of BALB/c (▪) and C57BL/6 mice (□). The difference in the fold change determined by qRT-PCR between BALB/c and C57BL/6 mice was not significant (*p* = 0.13 and 0.16, respectively).
(TIF)

Figure S2 **Growth curves of the** ***B. pseudomallei*** **K96243 wild-type (•) and** ***bprD*** **mutant (□) strains; no significant difference was evident (** ***p*** **= 0.23).**
(TIF)

Figure S3 **Numbers of bacteria in the spleen (A), lung (B), and liver (C) of BALB/c (♦) and C57BL/6 (▪) mice on days 3 to 5.**
(TIF)

Figure S4 **Schematic diagrams of the construction of the** ***B. pseudomallei*** **K96243** ***bprD*** **mutant using pDM4.** (A) Small arrows represent primer sites used to generate the upstream and downstream fragments to clone into pDM4 together with the Tc^r^ cassette from pUTminiTn5Tc, to generate the pDMΔ*bprD*::Tc^r^ plasmid. (B1) Dotted cross indicates the first and second recombination steps used to replace *bprD* on the *B. pseudomallei* K96243 chromosome with the Tc^r^ cassette from pDMΔ*bprD*::Tc^r^, resulting in generation of the *bprD* mutant. (B2) Dotted cross indicates the first and second recombination steps at the same site leading to abortive allelic exchange and generation of the wild type rather than the *bprD* mutant.
(TIF)
