# Supplementary figures and images for: Characterization of BPSS1521 (bprD), a Regulator of Burkholderia pseudomallei Virulence Gene Expression in the Mouse Model
Source: PLoS One. 2014 Aug 11;9(8):e104313. doi: 10.1371/journal.pone.0104313 (PMC4128674; doi:10.1371/journal.pone.0104313)

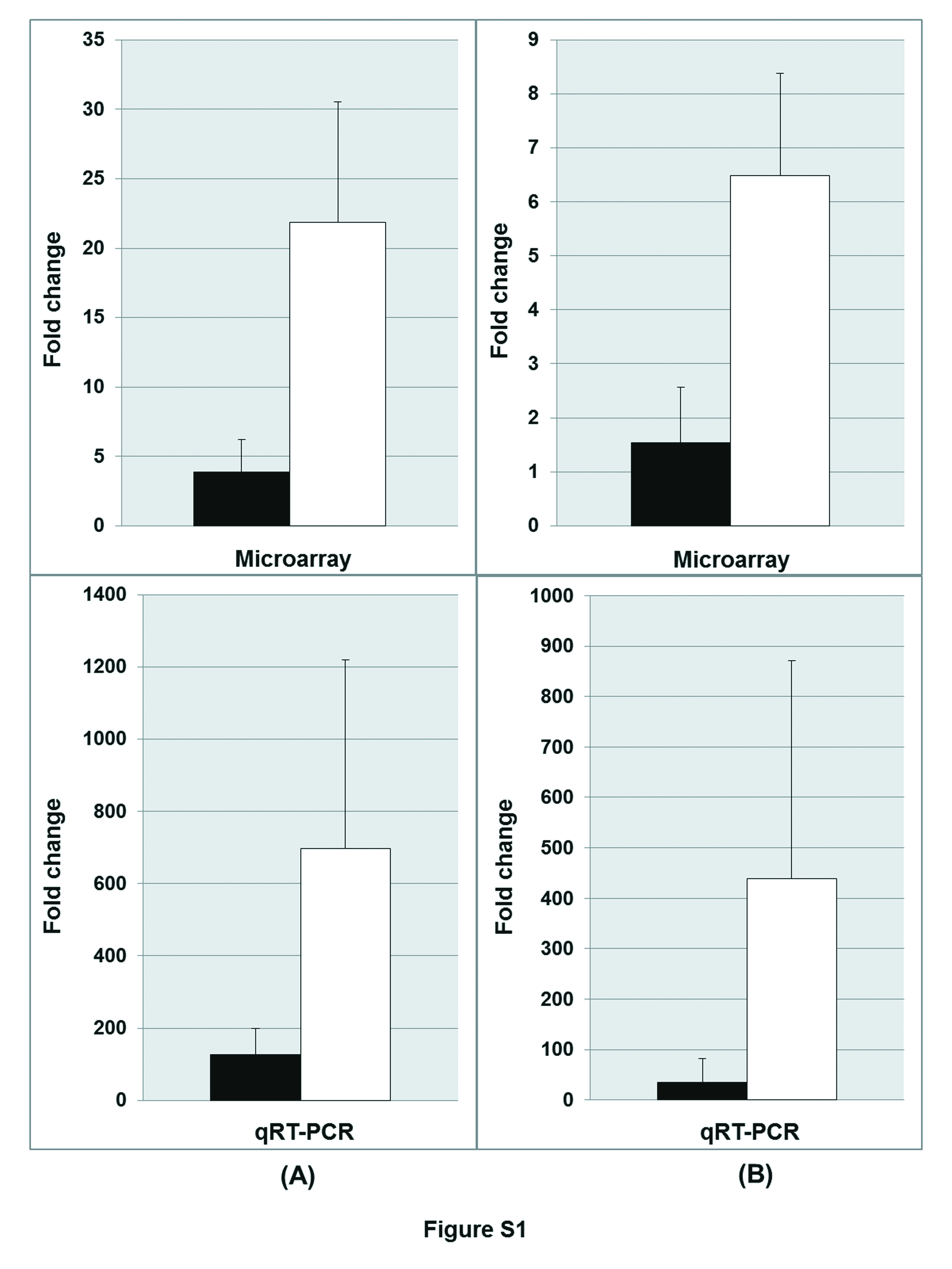

Supplement: Figure S1 — Fold changes in gene expression in vivo / in vitro . Fold changes in the expression of the BPSS1521 (A) and BPSS1512 (B) genes as determined by DNA microarray (upper) and validated by qRT-PCR (lower) in the lungs of BALB/c (▪) and C57BL/6 mice (□). The difference in the fold change determined by qRT-PCR between BALB/c and C57BL/6 mice was not significant (p = 0.13 and 0.16, respectively). (TIF) [file pone.0104313.s001.tif]

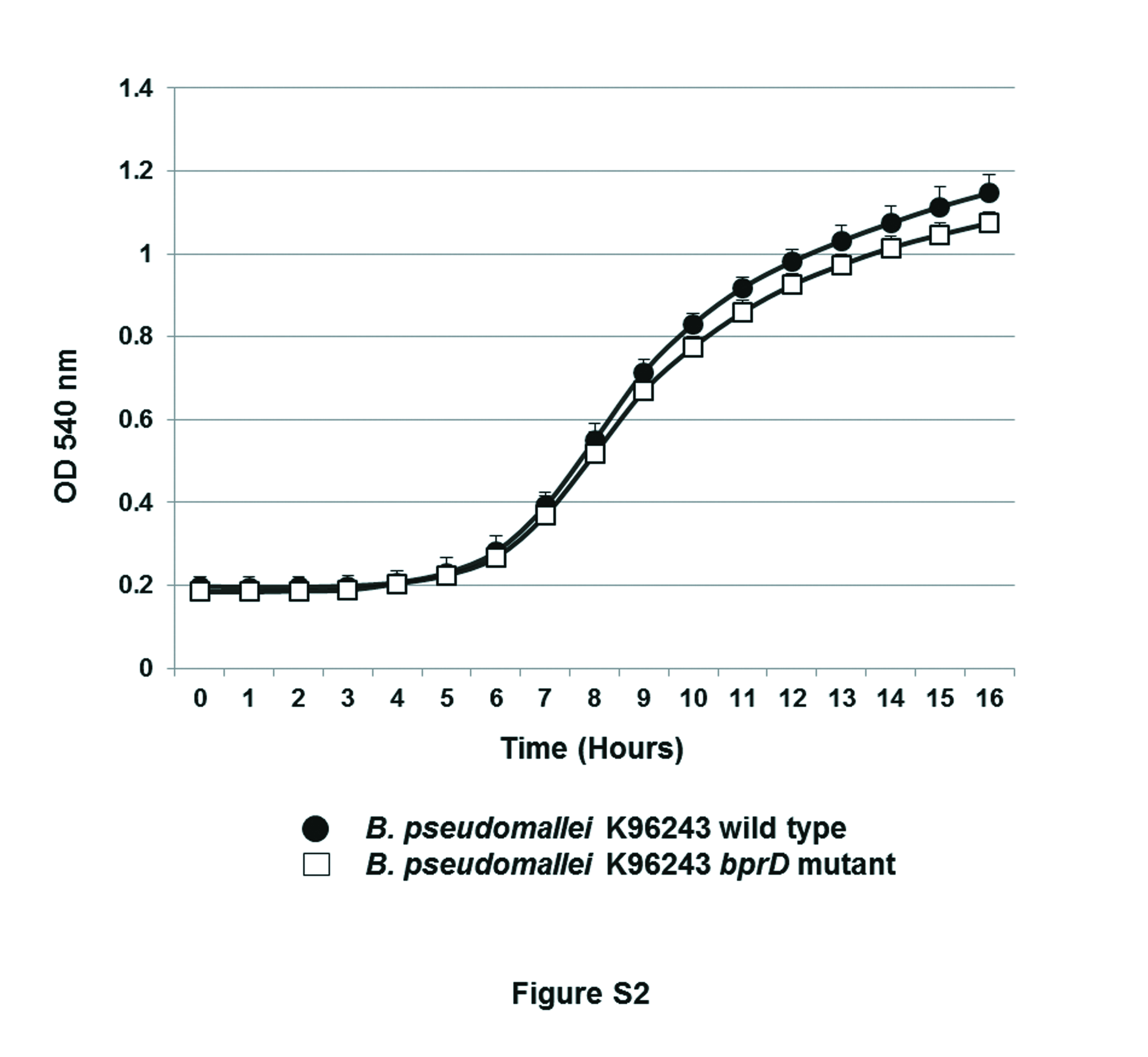

Supplement: Figure S2 — Growth curves of the B. pseudomallei K96243 wild-type (•) and bprD mutant (□) strains; no significant difference was evident ( p = 0.23). (TIF) [file pone.0104313.s002.tif]

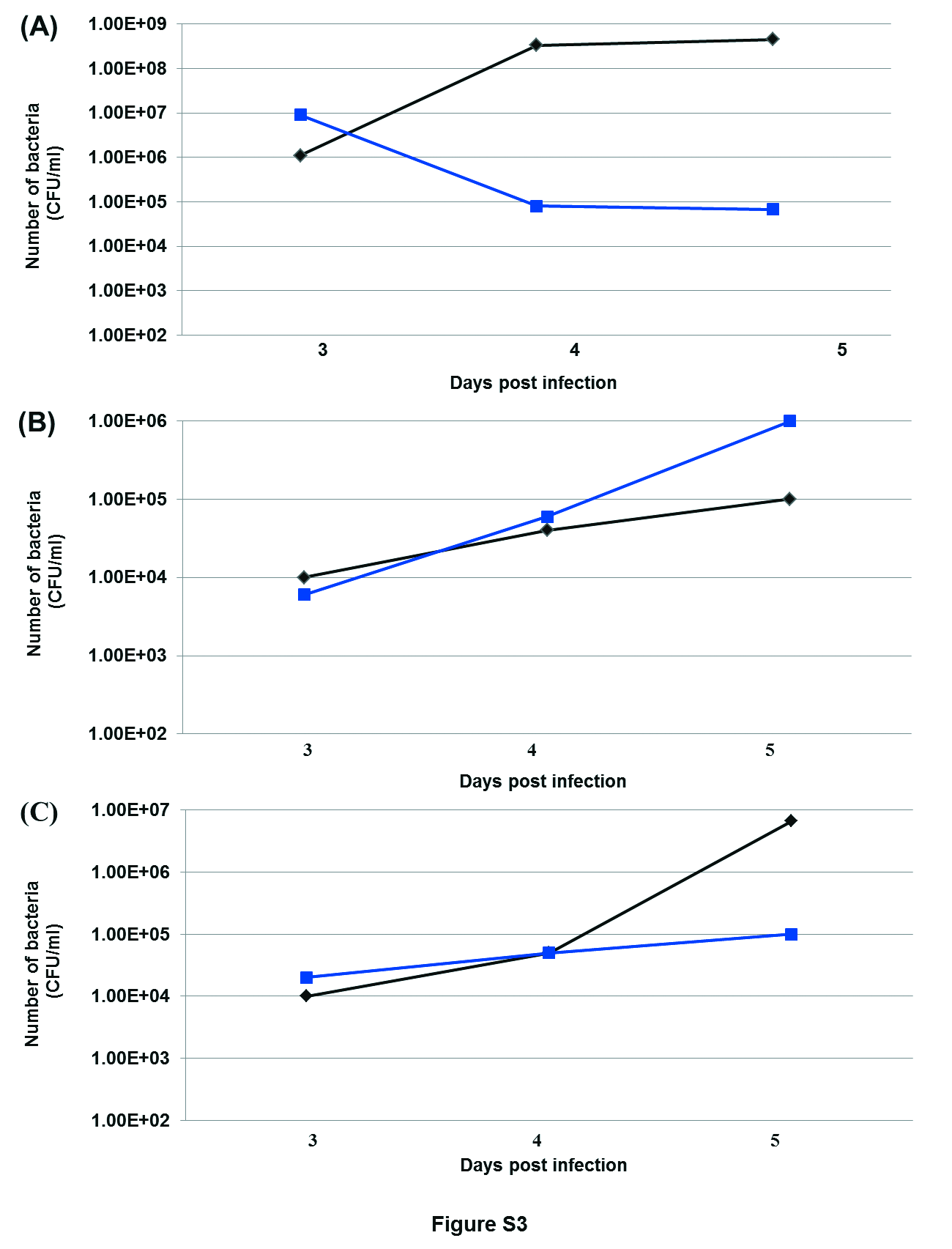

Supplement: Figure S3 — Numbers of bacteria in the spleen (A), lung (B), and liver (C) of BALB/c (♦) and C57BL/6 (▪) mice on days 3 to 5. (TIF) [file pone.0104313.s003.tif]

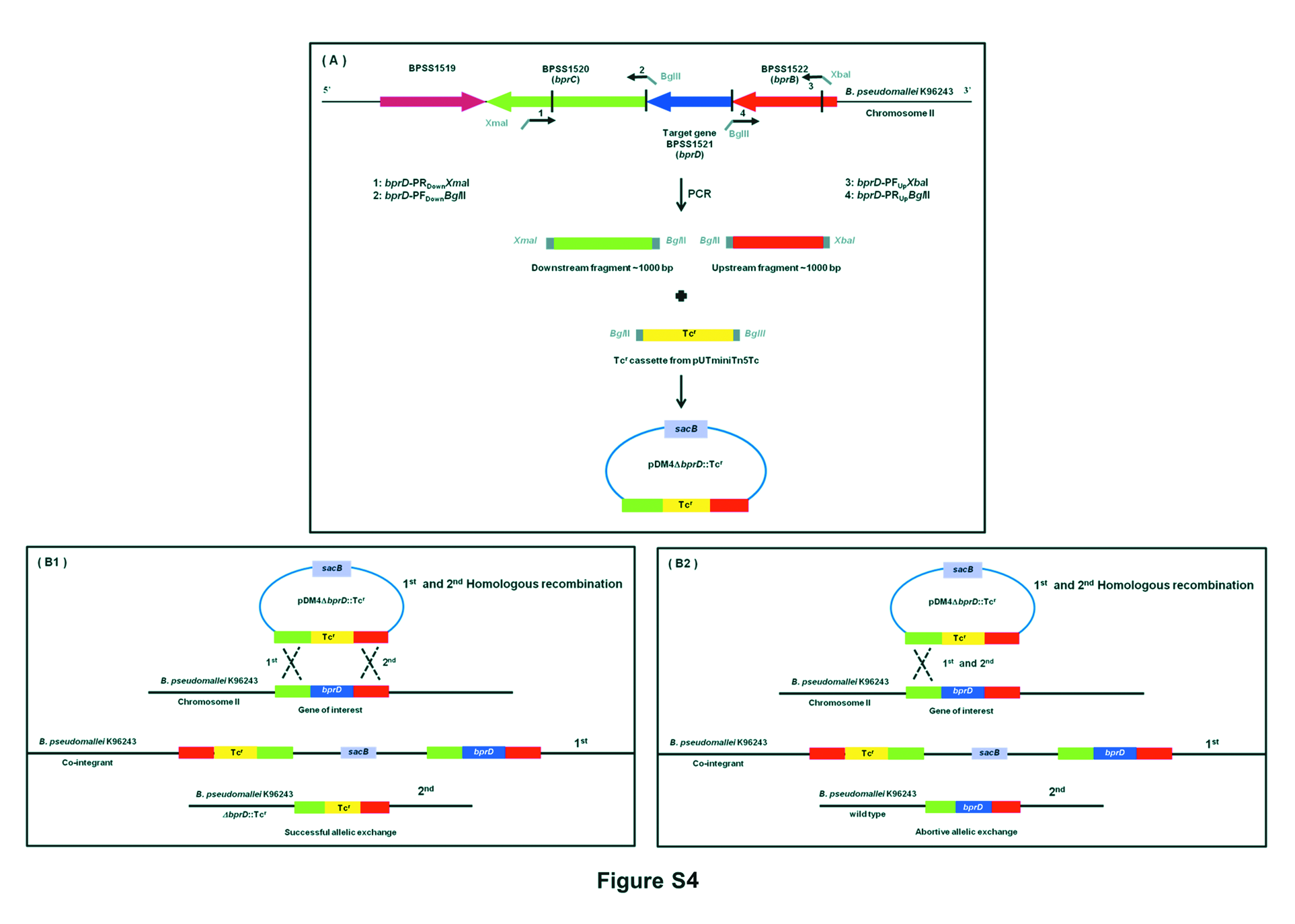

Supplement: Figure S4 — Schematic diagrams of the construction of the B. pseudomallei K96243 bprD mutant using pDM4. (A) Small arrows represent primer sites used to generate the upstream and downstream fragments to clone into pDM4 together with the Tcr cassette from pUTminiTn5Tc, to generate the pDMΔbprD::Tcr plasmid. (B1) Dotted cross indicates the first and second recombination steps used to replace bprD on the B. pseudomallei K96243 chromosome with the Tcr cassette from pDMΔbprD::Tcr, resulting in generation of the bprD mutant. (B2) Dotted cross indicates the first and second recombination steps at the same site leading to abortive allelic exchange and generation of the wild type rather than the bprD mutant. (TIF) [file pone.0104313.s004.tif]
